# Supplementary material for: In Vivo Phosphorylation of the Cytosolic Glucose-6-Phosphate Dehydrogenase Isozyme G6PD6 in Phosphate-Resupplied Arabidopsis thaliana Suspension Cells and Seedlings
Source: Plants (Basel). 2023 Dec 21;13(1):31. doi: 10.3390/plants13010031 (PMC10780934; doi:10.3390/plants13010031)
Supplement: Supplementary file 1 [file plants-13-00031-s001.zip › plants-2727721-supplementary.pdf]

Table S1. Protein accession numbers of cytosolic G6PD orthologs aligned in Figure 2.

G6PD ID	Species	Accession#
*At*G6PD1	*Arabidopsis thaliana*	NP_198428
*At*G6PD2	*Arabidopsis thaliana*	NP_196815
*At*G6PD3	*Arabidopsis thaliana*	NP_173838
*At*G6PD4	*Arabidopsis thaliana*	NP_563844
*At*G6PD5	*Arabidopsis thaliana*	NP_189366
*At*G6PD6	*Arabidopsis thaliana*	NP_198892
*An*G6PD	*Aspergillus niger*	P48826
*Ce*G6PD	*Caenorhabditis elegans*	Q27464
*Cv*G6PD1	*Chlorella vulgaris*	BAB96757
*Gm*G6PD2	*Glycine max*	XP_040868353
*Gm*G6PD4	*Glycine max*	XP_006599060
*Hs*G6PD	*Homo Sapiens*	P11413
*Hv*G6PD	*Hordeum vulgare*	XP_044966924
*Mm*G6PD	*Mus musculus*	Q00612
*Np*G6PD	*Nostoc punctiforme*	P48848
*Os*G6PD1	*Oryza sativa Japonica Group*	XP_015627059
*Os*G6PD2	*Oryza sativa Japonica Group*	XP_015635836
*Pp*G6PD1	*Physcomitrium patens*	XP_024357847
*Rn*G6PD	*Rattus norvegicus*	P05370
*Sl*G6PD4	*Solanum lycopersicum*	XP_004231802.1
*St*G6PD2	*Solanum tuberosum*	NP_001275397.1

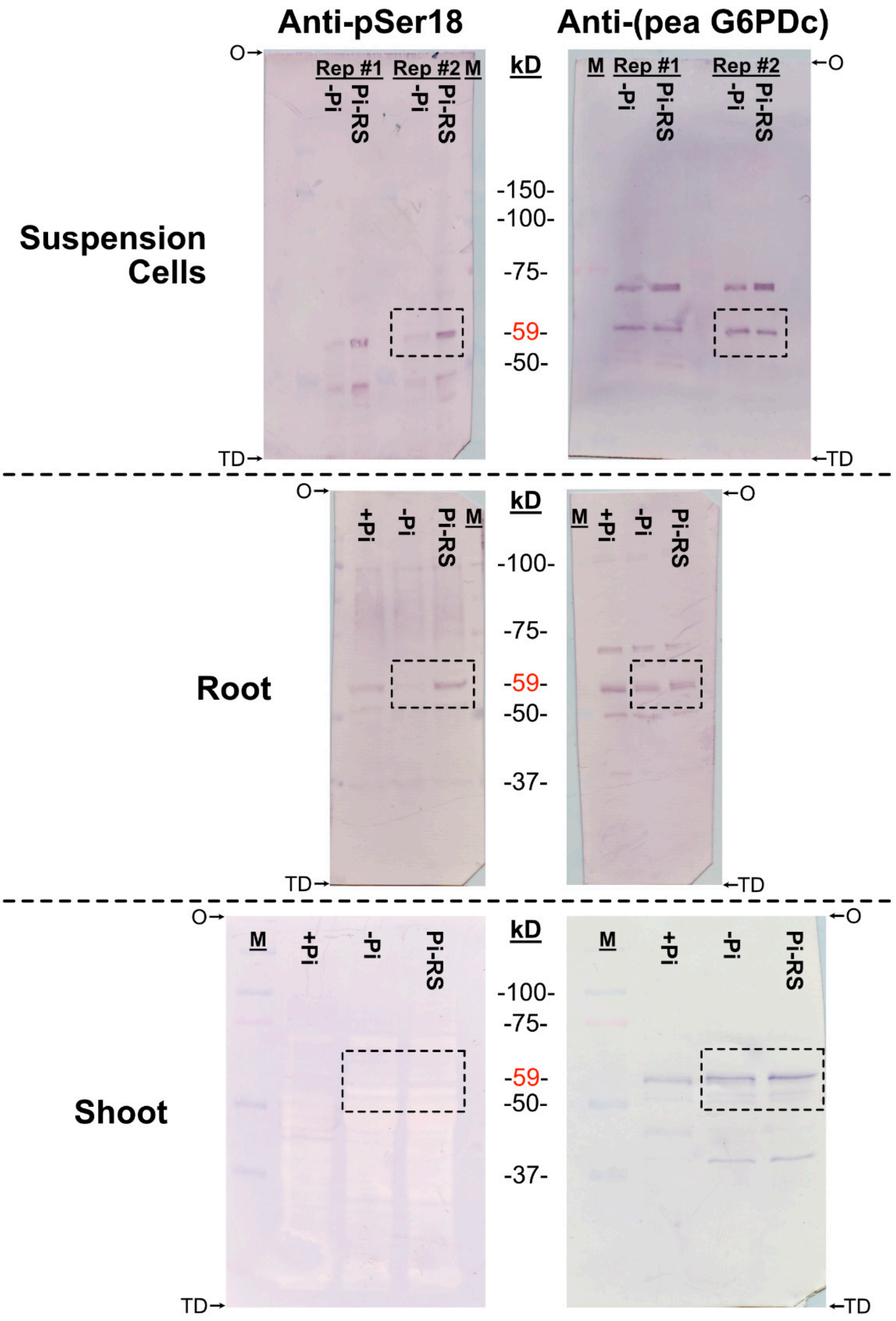

Figure S1. Uncropped immunoblots of Figure 6, showing impact of Pi-resupply on G6PD6's Ser18 phosphorylation status. Clarified extracts from: Pi-sufficient ('+Pi'), –Pi, and 48 h Pi-resupplied (Pi-RS) suspension cells, and -Pi or 48 h Pi-resupplied seedlings (shoots and roots) were subjected to SDS/PAGE followed by immunoblotting with anti-pSer18 (+10 µg/mL dephospho (deP)-peptide) or anti-(pea G6PDc). Approximately 8 and 1 µg of protein were loaded into each lane of the anti-pSer18 and anti-(pea G6PDc) immunoblots, respectively. Boxed areas represent the cropped portions shown in Figure 6. 'O' and 'TD' denote origin and tracking dye fronts, respectively, and 'M' indicates various pre-stained molecular mass standards.

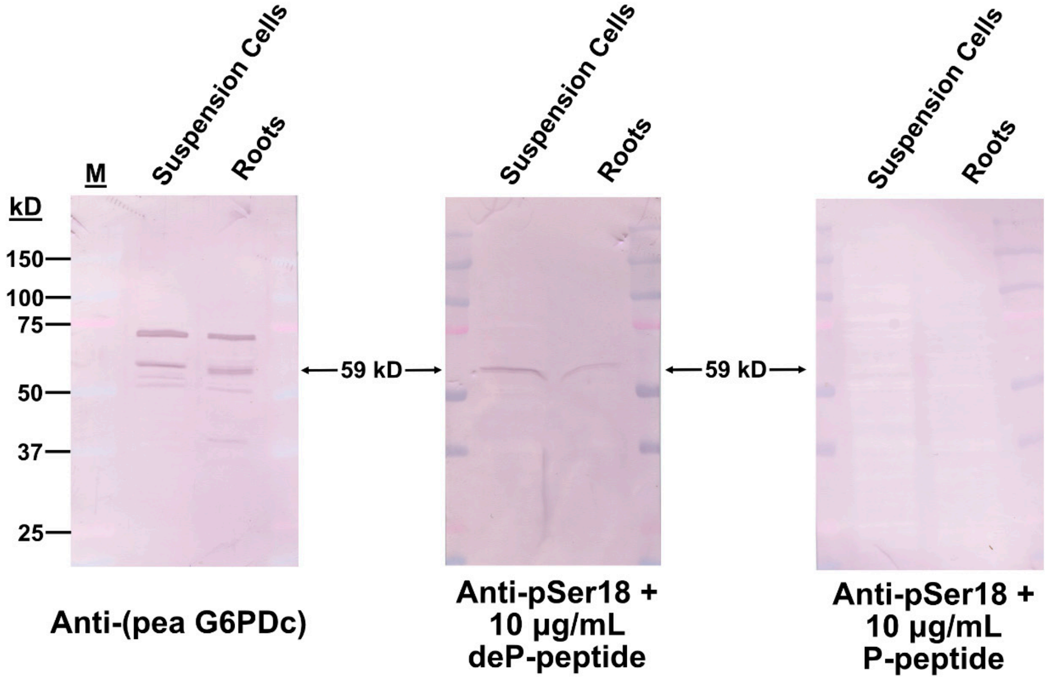

Figure S2. Specificity of phospho-site specific antibody raised against pSer18 of *Arabidopsis* G6PD6 (anti-pSer18). Clarified extracts from the 48 h Pi-resupplied suspension cells and seedlings (roots) were subjected to SDS/PAGE followed by immunoblotting with anti-(pea G6PDc), or anti-pSer18 in the presence of either 10 µg/mL of the dephospho- (deP-) or phospho- (P-) peptide shown in Figure 5A. Approximately 1 and 8 µg of protein were loaded into each lane of the anti-(pea G6PDc) and anti-pSer18 immunoblots, respectively.
